# Association of plasma soluble urokinase plasminogen activator receptor concentrations and migraine with aura: a REFORM study

**DOI:** 10.1093/braincomms/fcae475

**Published:** 2025-02-17

**Authors:** Betel Tesfay, Håkan Ashina, Rune Häckert Christensen, Haidar M Al-Khazali, William Kristian Karlsson, Faisal Mohammad Amin, Baker Nawfal Jawad, Ove Andersen, Messoud Ashina

**Affiliations:** Department of Neurology, Danish Headache Center, Copenhagen University Hospital—Rigshospitalet, Copenhagen 2600, Denmark; Department of Clinical Medicine, Faculty of Health and Medical Sciences, University of Copenhagen, Copenhagen 2200, Denmark; Department of Neurology, Danish Headache Center, Copenhagen University Hospital—Rigshospitalet, Copenhagen 2600, Denmark; Department of Clinical Medicine, Faculty of Health and Medical Sciences, University of Copenhagen, Copenhagen 2200, Denmark; Translational Research Center, Copenhagen University Hospital—Rigshospitalet, Copenhagen 2600, Denmark; Department of Neurology, Danish Headache Center, Copenhagen University Hospital—Rigshospitalet, Copenhagen 2600, Denmark; Department of Clinical Medicine, Faculty of Health and Medical Sciences, University of Copenhagen, Copenhagen 2200, Denmark; Translational Research Center, Copenhagen University Hospital—Rigshospitalet, Copenhagen 2600, Denmark; Department of Neurology, Danish Headache Center, Copenhagen University Hospital—Rigshospitalet, Copenhagen 2600, Denmark; Department of Clinical Medicine, Faculty of Health and Medical Sciences, University of Copenhagen, Copenhagen 2200, Denmark; Translational Research Center, Copenhagen University Hospital—Rigshospitalet, Copenhagen 2600, Denmark; Department of Neurology, Danish Headache Center, Copenhagen University Hospital—Rigshospitalet, Copenhagen 2600, Denmark; Department of Clinical Medicine, Faculty of Health and Medical Sciences, University of Copenhagen, Copenhagen 2200, Denmark; Department of Neurology, Danish Headache Center, Copenhagen University Hospital—Rigshospitalet, Copenhagen 2600, Denmark; Department of Clinical Medicine, Faculty of Health and Medical Sciences, University of Copenhagen, Copenhagen 2200, Denmark; Department of Clinical Medicine, Faculty of Health and Medical Sciences, University of Copenhagen, Copenhagen 2200, Denmark; Department of Clinical Research, Copenhagen University Hospital—Amager and Hvidovre, Hvidovre 2650, Denmark; Department of Clinical Medicine, Faculty of Health and Medical Sciences, University of Copenhagen, Copenhagen 2200, Denmark; Department of Clinical Research, Copenhagen University Hospital—Amager and Hvidovre, Hvidovre 2650, Denmark; Department of Emergency Medicine, Copenhagen University Hospital—Amager and Hvidovre, Hvidovre 2650, Denmark; Department of Neurology, Danish Headache Center, Copenhagen University Hospital—Rigshospitalet, Copenhagen 2600, Denmark; Department of Clinical Medicine, Faculty of Health and Medical Sciences, University of Copenhagen, Copenhagen 2200, Denmark; Danish Knowledge Center on Headache Disorders, Copenhagen University Hospital—Rigshospitalet, Copenhagen 2600, Denmark

**Keywords:** migraine, headache, biomarker, suPAR, inflammation

## Abstract

Soluble urokinase plasminogen activator receptor (suPAR) has garnered attention as a potential blood-based biomarker for low-grade chronic inflammation. However, its specific association with migraine, including its subtypes, remains to be elucidated. We sought to examine the association of plasma suPAR levels with migraine and its subtypes. In this single-centre, cross-sectional study, plasma was collected at a single time point in adults with migraine and sex-matched healthy controls from October 2020 to June 2022. The quantification of plasma suPAR levels was performed in a blinded fashion using a validated enzyme-linked immunosorbent assay. Plasma suPAR levels were compared between participants with migraine (including subgroups) and healthy controls. Plasma samples were analysed from 634 eligible participants with migraine [mean (SD) age, 44.0 (12.2) years; 568 (89.6%) females] and 154 healthy controls [mean (SD), 41.3 (11.8%) years; 132 (86%) females]. Plasma suPAR levels were 6.7% higher (95% CI: 0.1–13.6%; *P* = 0.045, adjusted for age, sex, body mass index and smoking) in participants with migraine *with* aura, when compared with healthy controls. Further analysis revealed no difference in plasma suPAR levels between the overall migraine group and healthy controls (3.7%; 95% CI: −0.7–8.2%; *P* = 0.097), as well as between participants with migraine without aura and healthy controls (2.5%; 95% CI: −2.9–8.3%; *P* = 0.81). Similarly, plasma suPAR levels did not differ across participants with episodic migraine, chronic migraine and healthy controls. Finally, we found no difference when comparing participants with migraine at time of blood sampling with participants with non-migraine headache (1.0%; 95% CI: −5.7–8.2; *P* > 0.99), participants without headache (1.2%; 95% CI: −4.2–7.0%; *P* > 0.99) or healthy controls (4.5%; 95% CI: −1.9–11.3%; *P* = 0.39). Elevated plasma suPAR levels in migraine with aura indicate the presence of low-grade chronic inflammation. Future research should explore the role of suPAR in the neurobiologic underpinnings of migraine with aura.

## Teaser

Tesfay *et al*. identified elevated plasma soluble urokinase plasminogen activator receptor levels in migraine with aura compared with healthy controls, suggesting low-grade chronic inflammation. This research highlights potential pathways involving microglial activation and endothelial dysfunction in migraine with aura, offering new insights for developing targeted mechanism-based treatments.

## Graphical Abstract

**Graphical Abstract:**
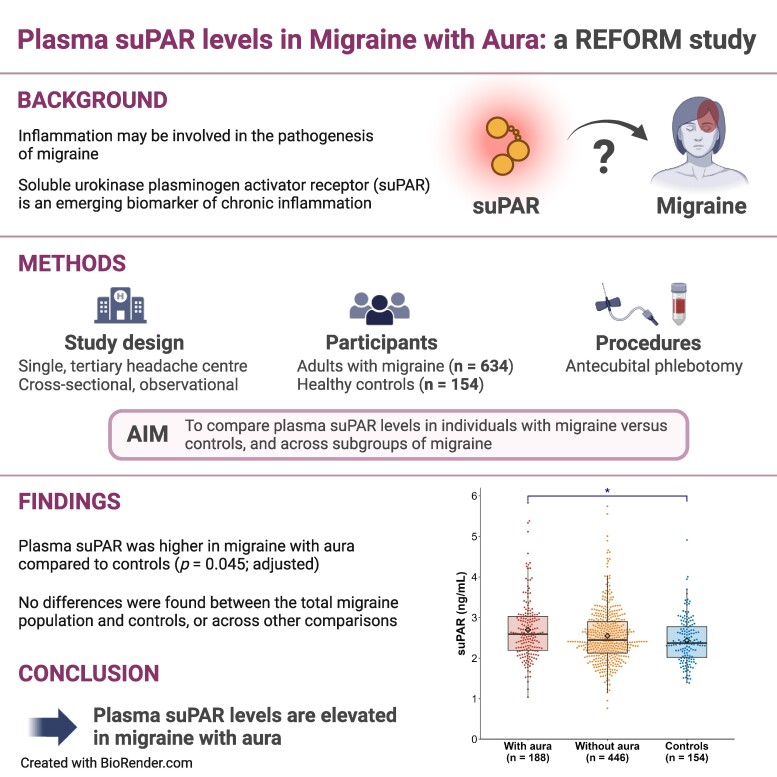

## Introduction

Migraine is a prevalent neurologic disorder and accounts for more years lived with disability than all other neurologic disorders combined.^1,2^ The clinical presentation is characterized by recurrent headache attacks of moderate or severe pain intensity and accompanying nausea, vomiting, photophobia and phonophobia.^3^ Furthermore, about one-third of those affected experience transient, fully reversible neurologic symptoms, referred to as migraine aura.^3^

While increasing evidence implicates inflammation in the meninges and cortical inflammation within the CNS as key pathophysiologic mechanisms in migraine,^4,5^ a definitive characterization of these inflammatory processes remains pending.^6^ In this context, soluble urokinase plasminogen activator receptor (suPAR) has emerged as a promising blood-based biomarker for understanding the low-grade chronic inflammation that might underlie migraine.^7^

suPAR, a stable circulating protein, is synthesized in response to inflammatory stimuli by cleaving the cell membrane–anchored urokinase plasminogen activator receptor (uPAR).^7,8^ The presence of uPAR across various immune cells, including microglia, macrophages, monocytes, T-lymphocytes, neutrophils and endothelial cells, underscores its importance in regulating a multitude of immune functions.^8,9^ Moreover, suPAR has been identified as an independent prognostic biomarker for various diseases associated with chronic inflammation and immune system activation, including cancer, cardiovascular disease, renal disease and Type 2 diabetes, as well as for mortality.^10-12^

To date, the exploration of suPAR in migraine has been limited to only one small case–control study involving 60 participants with migraine and 30 healthy controls.^13^ This study revealed elevated serum suPAR levels during the migraine attack (ictal) phase compared with the headache-free (inter-ictal) phase, with higher levels in people with migraine with aura (MA), as opposed to those with migraine without aura (MO). Given the limited sample size of this previous study, considerable gaps remain in the current understanding of blood levels of suPAR in people with migraine. To better characterize the profile of circulating suPAR levels in migraine, we therefore conducted the present study to compare plasma suPAR levels in a large sample of adult participants with migraine and healthy controls.

## Materials and methods

### Design

This was a single-centre, cross-sectional study conducted at the research facilities of the Department of Neurology, Danish Headache Center, Copenhagen University Hospital—Rigshospitalet. The data presented herein are derived from the parental Registry for Migraine (REFORM) study. A detailed description of the design and methods used has been published elsewhere.^14^ The participant enrolment spanned from October 2020 to June 2022.

All participants provided written informed consent prior to the commencement of any study-related procedures. The study protocol was approved by the Health Research Ethics Committee of the Capital Region of Denmark and the Danish Data Protection Agency. The parental REFORM study was also registered with ClinicalTrials.gov (identifiers: NCT04603976 and NCT046740202) and conducted in accordance with the principles of the Declaration of Helsinki.^14,15^ Moreover, our study adhered to the Strengthening the Reporting of Observational Studies in Epidemiology reporting guidelines.^16^

### Participants

For participants with migraine, recruitment primarily occurred at the outpatient clinic of the Danish Headache Center, while healthy controls were sourced through targeted web-based advertisements. All headache diagnoses were classified according to the third edition of the International Classification of Headache Disorders (ICHD-3).^3^ A full description of recruitment process and eligibility criteria for both participants with migraine and healthy controls has been published elsewhere.^14^

The main inclusion criteria for participants with migraine was a diagnosis of MO, MA or chronic migraine.^3^ The participants were required to be 18 years of age or above, report a history of migraine for at least 1 year prior to enrolment and experience an average of at least 4 monthly migraine days (MMDs) during the 3 months before enrolment. The use of preventive migraine medication, except for drugs-directed calcitonin gene-related peptide signalling, was allowed, provided the dosage had been stable for at least 2 months prior to enrolment. The main exclusion criteria were a history of post-traumatic headache, hemiplegic migraine or cluster headache, as well as the inability to distinguish migraine headache from other headache types. In addition, for this specific investigation, the participants were excluded if they reported concurrent use of immunosuppressive drugs.

The control group comprised adult healthy volunteers with no personal history of any headache disorder, except for infrequent episodic tension-type headache. Additional exclusion criteria included first-degree relatives with a primary headache disorder, except for tension-type headache occurring ≤5 days per month, as well as current or previous clinically significant medical conditions, including neurological and psychiatric disorders, or regular medication use.

### Procedures

#### Clinical data assessment

Upon study inclusion, the participants with migraine underwent an in-person semi-structured interview conducted by a physician. This interview recorded information on demographic and clinical characteristics, as well as medicines history. A more detailed description of the semi-structured interview has been reported elsewhere.^14^

At the time of blood sampling, additional information was recorded on current headache status and characteristics, accompanying symptoms and a detailed account of headache occurrences and medication use in the 72 h prior to blood sampling. For the healthy controls, the clinical data assessment included a brief in-person semi-structured interview focusing on demographic and lifestyle data.

#### Description of laboratory testing

Blood sampling was conducted on the day of each participant’s enrolment. Peripheral venous cannulation of the antecubital fossa was performed, and samples were collected in 9-mL dipotassium ethylenediaminetetraacetate tubes. These tubes were then centrifugated at 4°C, 2200*×g* for 10 min. Following the centrifugation, plasma samples were transferred into coded cryotubes and stored at −80°C until analysis.

The analysis of plasma suPAR levels took place between March and April 2023. Two experienced laboratory technicians, blinded to group assignment and clinical data, conducted the quantitative measurements of plasma suPAR levels using a validated and commercially available enzyme-linked immunosorbent assay kit (suPARnostic®, ViroGates A/S, Birkeroed, Denmark). This process was carried out in strict accordance with the manufacturer’s protocol. The intra- and inter-assay coefficients of variation were 2.2 and 2.3%, respectively. The suPARnostic® enzyme-linked immunosorbent assay has a lower detection limit of 0.4 ng/ml.

### Outcome measures

#### Classification of participants

**Figure 1:**
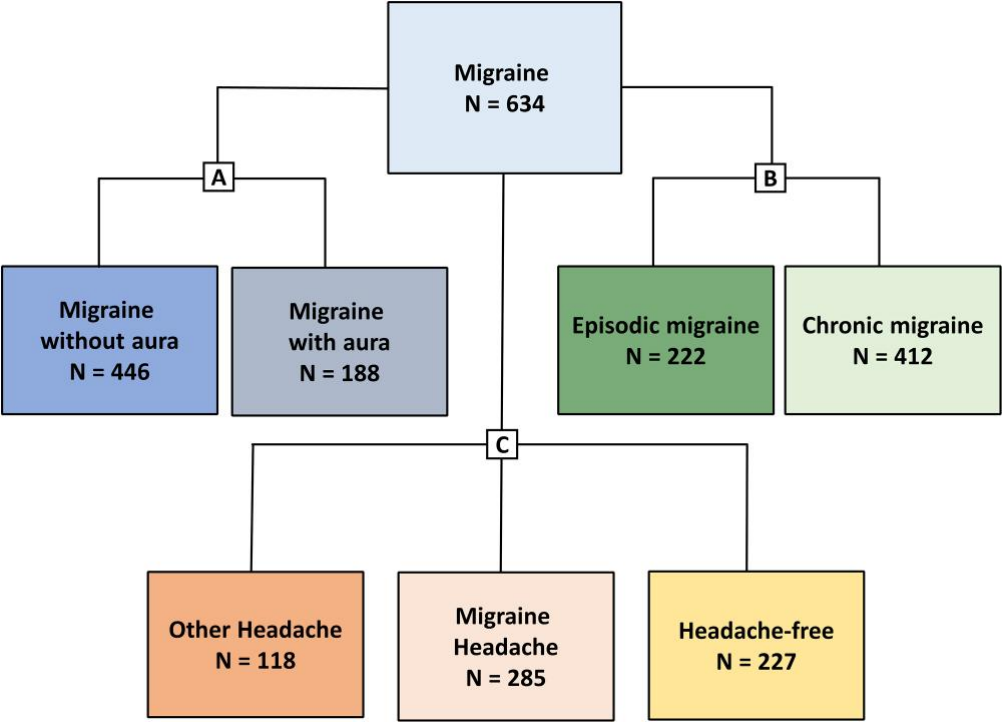
**Classification of participants with migraine.** The participants with migraine were categorized into three main groups. Each group was subsequently divided into specific sub-categories (**A**) based on the presence or absence of aura into two subgroups: MO and MA; (**B**) based on the frequency of migraine days into two subgroups: episodic migraine and chronic migraine and (**C**) based on the headache status at the time of blood sampling into three subgroups: migraine headache (ictal status), other headache and headache free.

The participants with migraine were assigned into three main categories, with each of these further divided into specific subgroups (Fig. 1). A non-mutually exclusive approach was used, allowing participants to belong to multiple subgroups simultaneously, thus enabling us to assess a broad spectrum of migraine subtypes in our study population.

The first category pertained to the presence or absence of aura, and the participants were divided into MO or MA subgroups. The latter subgroup consisted of participants diagnosed with exclusively MA or diagnosed with both MO and MA. The second category was established based on the frequency of migraine and headache, and the participants were divided into two subgroups: episodic migraine or chronic migraine. Of note, chronic migraine was classified in accordance with ICHD-3 as having at least 15 monthly headache days, with a minimum of eight meeting the criteria for a migraine headache, for a minimum period of 3 months.^3^ The third category pertained to the headache status at the time of blood sampling. The participants were divided into the following subgroups: migraine headache (ictal status), other headache and headache free. The migraine headache subgroup consisted of participants who were experiencing a headache that met the ICHD-3 criteria for definite migraine or probable migraine.^3^ The other headache subgroup consisted of participants who were experiencing a headache that did not fulfil the criteria for definite or probable migraine.

#### Outcomes

The primary outcome was the comparison of plasma suPAR levels between participants with migraine and healthy controls. The secondary outcomes included the comparison of plasma suPAR levels across the following subgroups: (i) MO, MA and healthy controls, (ii) episodic migraine, chronic migraine and healthy controls and lastly, (iii) ictal status, other headache status, headache-free status and healthy controls.

For the analysis of exploratory outcomes, we assessed the impact of the following variables on plasma suPAR levels: mean MMD, mean MMD with both aura and headache and mean monthly days with acute headache medication use. Furthermore, we assessed the impact of current use of preventive migraine medication and presence of medication-overuse headache.

### Statistical analysis

An overview of the statistical analyses is provided in Supplementary Table 1. The study aimed for a target enrolment of ≥600 participants with migraine, as previously defined.^14^ Categorical data were presented as counts and frequencies, whereas continuous data were summarized as mean ± standard deviation (SD) or median with interquartile range (IQR), contingent upon the data distribution. An exception was made for MMD, MMD with both aura and headache and monthly days with acute headache medication use, where mean values were reported in accordance with standard practices in migraine research. We assessed the data distribution using visual inspection of histograms and quartile–quartile plots. Demographic and characteristic comparisons across groups were conducted using unpaired *t*-test, Wilcoxon rank-sum test or Pearson’s ꭓ^2^ test.

Using linear regression modelling, plasma suPAR levels were compared pairwise between participants with migraine and healthy controls, as well as across clinically relevant subgroups. For the investigation of ongoing use of preventive migraine medications, we analysed participants with episodic and chronic migraine separately. All analyses were conducted: (i) unadjusted and (ii) adjusted for age, sex, body mass index and smoking status. A cut-off for plasma suPAR levels exceeding 6.0 ng/mL was applied. This threshold was based on findings from previous studies involving acute medical patients, where suPAR levels above 6.0 ng/mL were indicative of significant systematic inflammation and an elevated risk of 30- and 90-day mortality.^17^ Due to a skewed distribution of plasma suPAR residuals, natural log transformation was applied before statistical analysis; differences between groups were thus expressed as percent-wise differences after back transformation of the estimates and confidence intervals (CIs). For plots, untransformed suPAR levels were presented.

For participants with migraine, we also did univariate linear regression analyses with plasma suPAR level (log-transformed) as the dependent variable with each of the demographics and clinical characteristics presented in the tables as covariates. Covariates associated with plasma suPAR level (log-transformed) with a *P*-value <0.1 were included in a multivariable linear regression model. We ensured assumptions were met by assessing model diagnostics for both univariate and the multivariable models and variance inflation factors (values <5 were deemed acceptable) for the multivariable model.

Spearman’s rho was calculated to explore correlations between plasma suPAR levels and mean MMD, mean MMD with both aura and headache and mean monthly days with acute headache medication use. All statistical tests were conducted as two-sided tests, and *P*-values <0.05 were considered statistically significant. To control for multiple testing, the *P*-values of pairwise comparisons for each subgroup cluster were adjusted using the Bonferroni method. Complete case analysis was applied as missing data were <5% for the analyses. All statistical analyses were performed using R Statistical Software (v4.2.2; R Core team 2022).

## Results

### Participant characteristics

A total of 643 participants with migraine and 154 sex-matched healthy controls were enrolled in the study. SuPAR measurements from nine participants with migraine were excluded due to concurrent use of immunosuppressive drugs (*n* = 7) or plasma suPAR levels exceeding 6.0 ng/mL (*n* = 2). Thus, 634 participants with migraine provided data eligible for analyses. Table 1 outlines demographic and clinical characteristics of the participants and healthy controls. The characteristics were comparable between the two groups, except for the participants with migraine being of older age [mean (SD) age, 44.0 (12.2) versus 41.3 (11.8) years; *P=* 0.007]. Both groups were predominantly female, with no differences between them [568/634 (89.6%) versus 132/154 (85.7%); *P=* 0.20]. Among the 634 participants with migraine, 188 (29.7%) had MA and 412 (65.0%) had chronic migraine. For the overall migraine population, the mean (SD) MMD was 14.4 (6.9), and the mean (SD) MMD with both aura and headache was 4.0 (5.2). In the subgroup analysis of the MO and MA groups, we found comparable characteristics and comorbidities, except for a higher occurrence of asthma in the MA group compared with the MO group [40/446 (9.0%) versus 28/188 (14.9%); *P=* 0.035].

**Table 1:** Demographics and clinical characteristics of the study populations

Demographic characteristics	Migraine	Healthy controls	MO	MA	*P*-value^a^	*P*-value^b^
No. of participants	634	154	446	188	NA	NA
Age, mean (SD), years	44.0 (12.2)	41.3 (11.8)	43.8 (12.4)	44.4 (12.0)	**0.007**	0.68
Female sex, *n* (%)	568 (89.6%)	132 (86%)	397 (89%)	171 (91%)	0.20	0.57
Body mass index, mean (SD), kg/m^2^	25.1 (4.9)	24.7 (4.0)	25.0 (4.7)	25.4 (5.4)	0.68	0.82
Current smokers, *n* (%)	65 (10.4%)	20 (13.4%)	49 (11.1%)	16 (8.7%)	0.31	0.47
Clinical characteristics						
Chronic migraine	412 (65.0%)	NA	280 (62.8%)	132 (70.2%)	NA	0.083
Migraine and aura frequency, mean (SD)						
MMDs^c^	14.4 (6.9)	NA	14.3 (6.9)	14.7 (7.0)	NA	0.45
Monthly days with aura^c^	4.0 (5,2)	NA	NA	4.0 (5,2)	NA	NA
Monthly days with use of acute medications	11.8 (6.5)	NA	11.9 (6.3)	11.6 (6.7)	NA	0.46
Comorbidities, *n* (%)						
Asthma	68 (10.7%)	NA	40 (9.0%)	28 (14.9%)	NA	**0.035**
Auto-immune conditions	69 (10.9%)	NA	47 (10.5%)	22 (11.7%)	NA	0.68
Daily neck pain (≥3 months)	96 (15.1%)	NA	28 (14.9%)	68 (15.2%)	NA	>0.99
Daily lumbar pain (≥3 months)	61 (9.6%)	NA	15 (8.0%)	46 (10.3%)	NA	0.46
Hypertension	69 (10.9%)	NA	54 (12.1%)	15 (8.0%)	NA	0.16
Other cardiovascular conditions	39 (6.2%)	NA	25 (5.6%)	14 (7.4%)	NA	0.37
History of cancer^d^	32 (5.1%)	NA	22 (4.9%)	10 (5.3%)	NA	0.84
Depression	62 (9.8%)	NA	43 (9.6%)	19 (10.1%)	NA	0.88
Anxiety	62 (9.8%)	NA	43 (9.6%)	19 (10.1%)	NA	0.88

*P*-values <0.05 are highlighted in bold.

NA, not applicable.

^a^Migraine versus healthy controls.

^b^Migraine without aura versus MA.

^c^Mean over 3 months period prior to study visit.

^d^With no current cancer diagnosis.

Table 2 provides an overview of headache status at the time of blood sampling and concurrent medication use among participants with migraine. Of the 634 participants, 318 (50.2%) used preventive migraine medication, and 220 (34.7%) fulfilled the criteria for medication-overuse headache. Data regarding headache status were available from 630 participants with migraine during blood sampling. Among these, 285 (45.2%) participants reported either a migraine attack (*n* = 162) or a probable migraine attack (*n* = 123). Furthermore, 118 (18.7%) participants experienced non-migraine headache, and 227 (36.0%) were headache free. In addition, the MO group had a higher intake of triptans ≤72 h prior to blood sampling compared with the MA group [156/446 (38.3%) versus 49/188 (28.3%); *P=* 0.023].

**Table 2:** Headache and treatment status at blood sampling

	Migraine	MO	MA	*P*-value^a^
Headache status, *n* (%)^b^	634	446	188	0.96
Migraine headache	285 (45.2%)	199 (44.8%)	86 (46.0%)	
Definite migraine	162 (25.7%)	112 (25.2%)	50 (26.7%)	
Probable migraine	123 (19.5%)	87 (19.6%)	36 (19.3%)	
Other headache	118 (18.7%)	85 (19.1%)	33 (17.6%)	
Headache free	227 (36.0%)	160 (36.0%)	68 (36.4%)	
Medication-overuse headache, *n* (%)	220 (34.7%)	154 (34.5%)	66 (35.1%)	0.93
Concurrent preventive medication, No. (%)^c^	318 (50.2%)	227 (50.9%)	91 (48.4%)	0.60
Concurrent use of statins, *n* (%)	27 (4.3%)	16 (3.6%)	11 (5.9%)	0.20
Intake of NSAID^d^	67 (11.7%)	49 (12.2%)	18 (10.5%)	0.37
Intake of triptans^d^	205 (35.3%)	156 (38.3%)	49 (28.3%)	**0**.**023**

*P*-values <0.05 are highlighted in bold.

NA, not applicable; NSAID, non-steroidal anti-inflammatory drugs.

^a^Migraine with aura versus MO.

^b^Headache status at time of blood sampling.

^c^Preventive medication within ≤3 months prior to study visit.

^d^Intake of medication ≤72 h prior to blood sampling.

### Plasma soluble urokinase plasminogen activator receptor levels in participants with migraine and in migraine subgroups

**Figure 2:**
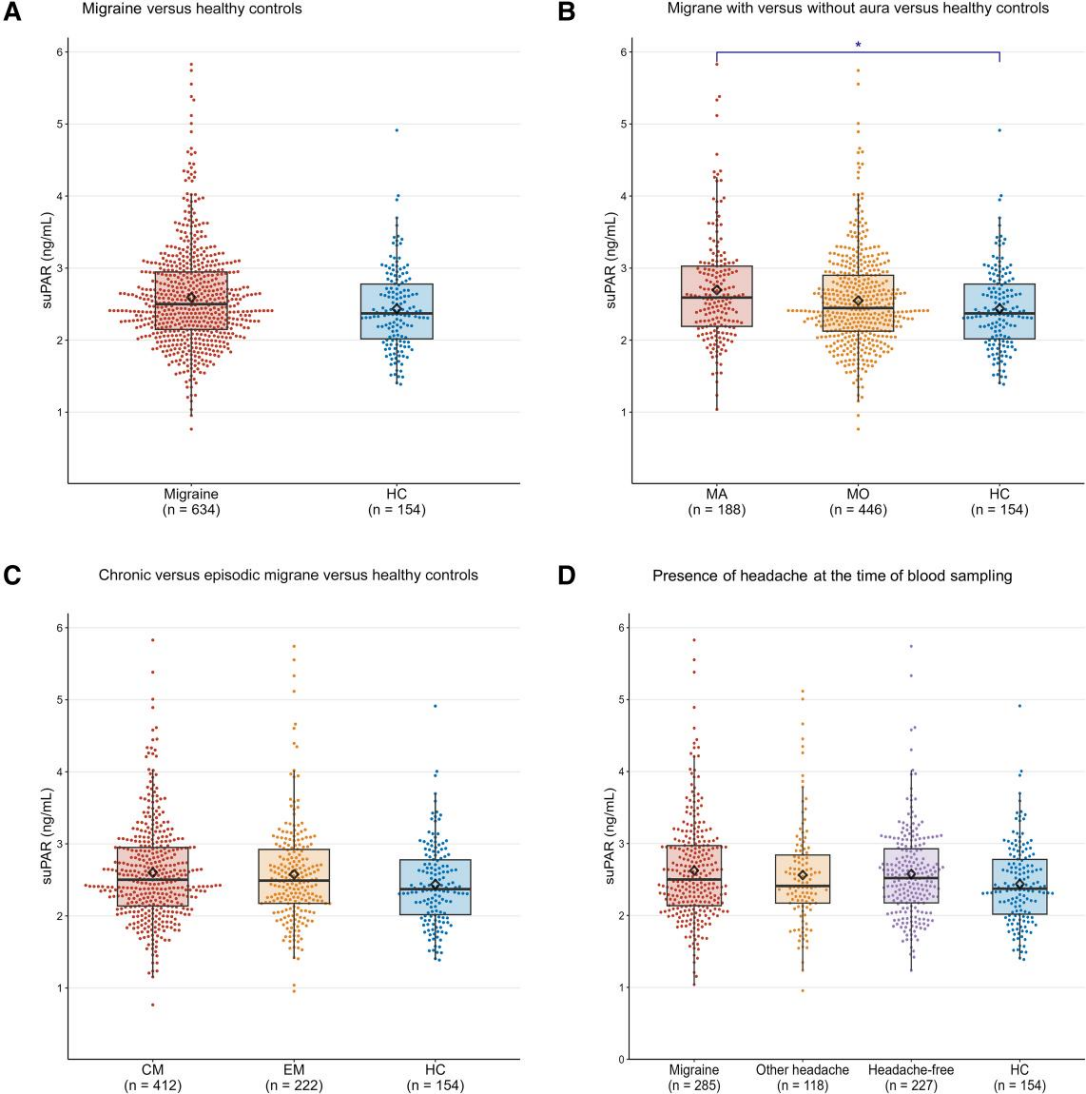
**Plasma suPAR levels in participants with migraine (including subgroups) and healthy controls.** Plasma suPAR levels in (**A**) migraine group and healthy controls (HCs); (**B**) the MA group, MO group and HC; (**C**) chronic migraine (CM) group, episodic migraine (EM) group and HC and (**D**) migraine headache group (migraine), other headache group or headache-free group, compared with HC. Box plots depict median (horizontal bar), IQR (hinges), and 1.5 × IQR (whiskers). Individual measurements of suPAR are represented by dots. *P-values <0.05. Statistical comparisons were conducted using linear regression, adjusted for age, sex, body mass index and smoking, with Bonferroni correction for multiple testing.

The plasma suPAR levels in both the migraine and control population are presented in Supplementary Table 2 and Fig. 2. The estimated relative differences (%) in plasma suPAR levels for each pairwise comparison are furthermore shown in Table 3, with all findings adjusted for age, sex, body mass index and smoking status.

**Table 3:** Estimated relative differences in plasma suPAR

	Unadjusted model		Adjusted model^a^	
	Estimated relative difference (%; 95% CI)	*P*-value	Estimated relative difference (%; 95% CI)	*P*-value
Overall comparison				
All participants with migraine versus HC	5.5 (0.9–10.3)	**0**.**018**	3.7 (−0.7–8.2)	0.097
History of aura				
MA versus without aura	5.4 (0.03–11.1)	**0**.**048**	4.0 (−1.1–9.4)	0.18
MA versus HC	9.5 (2.6–17.0)	**0**.**003**	6.7 (0.1–13.6)	**0**.**045**
MO versus HC	3.9 (−1.8–9.9)	0.31	2.5 (−2.9–8.3)	0.81
Chronic migraine				
Chronic versus episodic migraine	1.0 (−3.9–6.3)	>0.99	0.7 (−4.0–5.7)	>0.99
Chronic migraine versus HC	5.9 (0.03–12.2)	**0**.**048**	4.0 (−1.6–9.9)	0.27
Episodic migraine versus HC	4.8 (−1.6–11.7)	0.22	3.2 (−2.9–9.7)	0.65
Ictal status				
Migraine versus other headache	2.7 (−4.4–10.4)	>0.99	1.0 (−5.7–8.2)	>0.99
Migraine versus headache free	1.1 (−4.7–7.2)	>0.99	1.2 (−4.2–7.0)	>0.99
Migraine versus HC	6.7 (−0.1–14.0)	0.057	4.5 (−1.9–11.3)	0.39
Other headache versus headache-free	−1.6 (−8.7–6.0)	>0.99	0.2 (−6.7–7.6)	>0.99
Other headache versus HC	3.8 (−4.2–12.6)	>0.99	3.5 (−4.2–11.8)	>0.99
Headache free versus HC	5.5 (−1.5–13.1)	0.23	3.2 (−3.3–10.3)	>0.99

*P*-values <0.05 before and after adjustment using the Bonferroni method are highlighted in bold.

HC, healthy controls.

^a^Adjusted for age, sex, body mass index and smoking.

We found no difference in plasma suPAR levels between participants with migraine and controls (3.7%; 95% CI: −0.7–8.2%; *P=* 0.097). The subgroup analyses revealed a 6.7% higher plasma suPAR concentration in MA participants, compared with controls (95% CI: 0.1–13.6%; *P=* 0.045). Conversely, no significant differences were detected in plasma suPAR levels between MO participants and healthy controls (2.5%; 95% CI: −2.9–8.3%; *P* = 0.81) or between MA and MO subgroups (4.0%; 95% CI: −1.1–9.4%; *P* = 0.18). Furthermore, we observed no significant differences in plasma suPAR levels between those with episodic migraine, chronic migraine and healthy controls (Table 3). The pairwise analyses also revealed no differences in plasma suPAR levels when comparing participants with ictal status, other headache, headache-free status and healthy controls (Fig. 2B).

### Impact of migraine and aura frequency, acute and preventive medication use, and medication overuse headache

Plasma suPAR levels did not correlate with MMD (*n* = 634; *r_s_*: 0.048, *P=* 0.23), MMD with both aura and headache (*n* = 188; *r_s_*: −0.021, *P=* 0.78) or monthly days with acute headache medication use (*n* = 632; *r_s_*: 0.052, *P=* 0.19). Furthermore, no significant differences in plasma suPAR levels were observed when comparing participants with episodic or chronic migraine who were on preventive migraine medication(s), those not on such treatments and healthy controls (Supplementary Table 3 and Fig. 1). Similarly, comparisons of plasma suPAR levels in participants with medication-overuse headache against those without such headache, and healthy controls, also revealed no significant differences (Supplementary Table 3 and Fig. 1).

### Multivariable linear regression analysis

Results of the univariate and multivariable linear regression analyses are available in Supplementary Table 4. In multivariable linear regression analysis, including participants with migraine with complete data (*n* = 623; 98.3%), the following characteristics were independently associated with plasma suPAR: age (0.4% increase per year; 95% CI: 0.3–0.6%; *P* < 0.0001), sex (10.1% higher in females compared to males; 95% CI: 10.3–17.2%; *P* = 0.003), body mass index (1.1% increase per kg/m^2^; 95% CI: 0.7–1.5%; *P* < 0.0001), active smoking (9.5% higher compared to non-smokers; 95% CI: 2.8–16.7%; *P* = 0.005), daily low back pain (7.7% higher if present; 95% CI: 0.8–15.1%; *P* = 0.028) and MA (4.4% higher compared with MO; 95% CI: 0.1–8.8%; *P* = 0.046).

## Discussion

In this cross-sectional study, higher plasma suPAR levels were observed in MA participants compared with healthy controls. Conversely, suPAR levels in MO participants were similar to those in healthy controls. No significant differences were noted between MA and MO subgroups for our primary analyses; however, multivariable linear regression analysis showed a significantly higher level in the MA subgroup when also adjusting for other relevant factors. No associations were detected between plasma suPAR levels and migraine frequency (episodic and chronic migraine) or headache status at the time of blood sampling (ictal, other headache and headache free). The elevated plasma suPAR levels indicate the presence of low-grade chronic inflammation in people with MA. Further insights into the molecular intricacies between inflammation and MA might provide clues into the neurobiologic basis of aura and pave the way for developing mechanism-based treatments.

To date, research on suPAR levels in migraine remains sparse, with data only available from one small study.^13^ This investigation reported elevated serum suPAR levels during the ictal phase in 30 MA participants, compared with 30 MO participants. Furthermore, serum suPAR levels were found to be higher during the ictal phase, without regards of MA presence, when compared with both the inter-ictal phase and healthy controls. Given the chronic nature of suPAR, featuring temporally stable blood levels minimally affected by short-term changes,^18,19^ it seems more conceivable to find similar levels between participants in the ictal phase, when compared with the inter-ictal phase. The discrepant findings between our investigation and this earlier study might be attributable to the inter-individual comparisons conducted within a small sample size, possibly amplifying the effect of sample variation.^20^

The observed findings suggest a specific association of elevated plasma suPAR levels with MA, without a similar elevation in those with MO or in correlation with headache or aura frequency. This presents an intriguing pattern that might point towards distinct pathophysiologic mechanisms underlying MA. In the following, we discuss the possibilities of suPAR being a marker of CNS inflammation, endothelial dysfunction or vascular health in MA.

### Microglial and macrophage-mediated inflammation in migraine with aura

**Figure 3:**
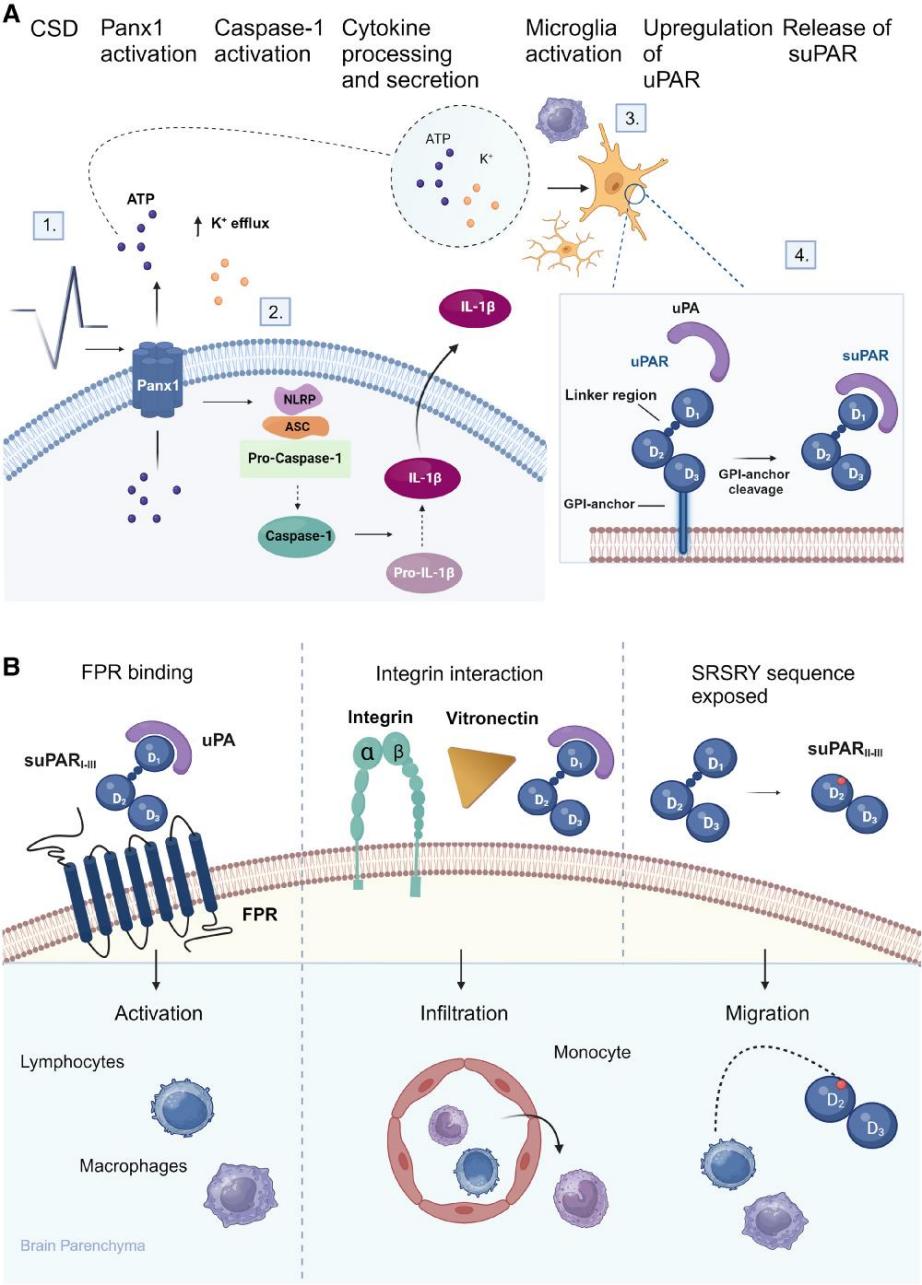
**Possible role of suPAR in microglial and macrophage-mediated inflammation in relation to MA.** (**A**) 1. CSD induces transient yet considerable alterations in the cerebral ionic environment, including elevated extracellular potassium ions (K^+^) and ATP. This may be partly facilitated by the rapid opening of mechanosensitive Pannexin 1 channels (Panx1). 2. The extracellular ATP stimulate and regulate the assembly of inflammasome complexes including nucleotide-binding oligomerization domain-like receptor protein (NLRP) and adaptor protein apoptosis-associated speck-like protein containing a CARD (ASC), which result in the autocatalytic activation of Caspase-1 and consequently the maturation and release of caspase-1 target molecules such as interleukin-1β (IL-1β). 3. The change in ionic extracellular environment may also lead to the stimulation and activation of resident microglial cells and macrophages. 4. Upon activation, microglia might upregulate the expression of the membrane-anchored uPAR. At the cell surface, upon inflammatory stimuli, suPAR can be generated through the cleavage of uPAR at the lipid glycosylphosphatidylinositol (GPI) anchor by a variety of proteases, such as urokinase plasminogen activator (uPA). Upregulation and cleavage of uPAR result in amplified release of suPAR into the bloodstream. (**B**) The suPAR–uPA complex binds to specific surface receptors, such as formyl peptide receptors (FPRs), regulating the activation and proliferation of immune cells. The suPAR–uPA complex also interacts with the extracellular adhesive glycoprotein vitronectin and adhesion receptors Integrin β1 and β2. These interactions facilitate the migration and infiltration of immune cells into the brain parenchyma. Upon cleavage of a full-length suPAR I–III at the linker region, the chemotactically active SRSRY motif on suPAR II–III is exposed, which in turn promotes immune cell migration. Figure created with BioRender.com.

In recent years, there has been a notable shift towards recognizing the active involvement of glial cells in migraine pathophysiology, departing from the predominant focus of basic research on migraine traditionally revolving around neuronal mechanisms.^21^ Below, we delineate several molecular connections linking suPAR with key components involved in the glial-mediated inflammation in MA (Fig. 3).

The neurophysiological correlate of migraine aura is widely believed to be cortical spreading depression (CSD), a slow propagating wave of neuronal and glial depolarization across the cerebral cortex, followed by transient suppression of cortical synaptic activity.^22^ Animal models of MA have demonstrated that CSD exerts a pro-inflammatory effect in the CNS parenchyma.^23,24^ This effect is purportedly initiated by the rapid opening of mechanosensitive Pannexin 1 channels and activation of Caspase-1, leading to the release of various molecules with potent noxious properties (e.g. potassium ions, protons, adenosine triphosphate and glutamate).^25-31^ This transient yet considerable alteration of the cerebral ionic environment is thought to stimulate and activate resident microglial cells and macrophages.^32^ Upon activation, microglia can elicit a signal transduction cascade by releasing pro-inflammatory cytokines such as tumour necrosis factor-α and interleukin-1β,^24,33-35^ both of which are elevated in MA participants and following CSD.^36,37^ This neuroinflammatory signalling cascade might ultimately manifest as chronic low-grade inflammation in cortical regions associated with aura, potentially mediated by glutamate-induced excitotoxicity or induced secretion of calcitonin gene-related peptide from trigeminal ganglion cells.^38-40^ Concurrently, these pro-inflammatory cytokines are implicated in the activation and sensitization of perivascular meningeal nociceptive afferents with the ensuing persistent neuronal discharge, possibly sustaining an ongoing migraine attack.^22,33,41^ Neuroimaging studies also provide support for increased glial-mediated inflammatory activity in participants with MA during inter-ictal state,^42,43^ specifically within regions important for CSD generation, such as the visual cortex, as well as in nociceptive processing structures (e.g. thalamus, primary and secondary somatosensory cortices and insula).^42^

Connecting suPAR to microglia, upregulated uPAR expression has been identified in murine resident microglial cells during conditions of both acute and chronic inflammation.^8^ This pattern is also observed in human studies across various neurological diseases, including traumatic brain injury, multiple sclerosis and Alzheimer’s disease.^44-47^ These findings suggest that elevated suPAR levels in participants with MA may be attributed to increased expression of uPAR triggered by CSD-induced activation of immune cells, notably microglia. This results in amplified release of suPAR into the bloodstream,^7^ with suPAR levels potentially reflecting the extent of microglial activity in participants with MA. SuPAR itself, along with its key ligand urokinase plasminogen activator, acts as a circulating protease.^19^ It regulates the activation and proliferation of various immune cells (e.g. monocytes, macrophages and glial cells) by binding to specific surface receptors, such as formyl peptide receptors and β2 integrins. suPAR also facilitates the migration and infiltration of leucocytes,^19,45^ partly through its complex interactions with the adhesive glycoprotein vitronectin and adhesion receptors of the integrin family (e.g. β1 and β2 integrins),^9,48^ as well as through its intrinsic chemotactic properties mediated by the chemotactically active SRSRY motif.^7,11^ These complex interrelated immunological properties of suPAR propose a pathogenic involvement of elevated suPAR levels in MA pathophysiology.

Finally, we must also consider the role of meningeal inflammation in MA pathophysiology. It has been suggested that activated microglia and the pro-nociceptive substances released into the cortical interstitium following CSD might migrate from the CNS parenchyma to access overlying pain-sensitive meninges.^25,49,50^ The transportation might occur along the perivascular pathways via bulk diffusion or through extracellular CSF movement in the glymphatic system, perhaps facilitated by chemotactic signals and inflammatory mediators.^22,51^ Previous data also demonstrate CSD-induced activation of dural and subdural macrophages, and the possible upregulation of uPAR expression on macrophages with subsequent secretion of pro-inflammatory mediators could promote local inflammation.^22,23,52^ Consistent with this, findings from a second neuroimaging study demonstrated inflammatory activity in the occipital parameningeal tissue in participants with visual aura.^49^

### Endothelial dysfunction in migraine with aura

Another potential explanation for the elevated suPAR levels in MA is endothelial dysfunction. In response to CSD, endothelial cells upregulate the expression of various inflammatory mediators,^53^ perhaps also including uPAR. The cleavage of membrane-bound uPAR then results in increased circulating levels of suPAR, which can serve as a marker of the endothelial dysfunction associated with CSD.^7^ In this context, it is worth considering that suPAR itself can contribute to disruption of the endothelial layer,^54^ leading to increased vascular permeability. This effect might be important since the endothelium acts as a barrier regulating the exchange of substance between the bloodstream and brain tissue.^55^ Extravasation and oedema can thus occur after blood–brain barrier disruption.^56^ This might allow immune cells (e.g. microglia) and inflammatory mediators to extravasate into brain tissue, contributing to low-grade chronic inflammation. In support, recent neuroimaging findings have found evidence of cortical inflammation within the visual cortices of exclusively people with MA,^42^ potentially ascribed to oedema. However, caution is warranted in attributing elevated suPAR levels in MA solely to endothelial dysfunction, as some neuroimaging data suggest that the blood–brain barrier remains intact in people with MA.^57,58^

### Vascular health indicator in migraine with aura

Elevated plasma suPAR levels have been associated with various vascular diseases.^10^ In the context of migraine, suPAR might be an indicator of the unique vascular risk profile that has been linked to MA. This could entail that circulating suPAR levels reflect endothelial dysfunction unrelated to CSD, as our results showed no correlation with aura frequency. Of note, epidemiologic studies have revealed an increased risk of ischaemic stroke in persons with MA.^59,60^ This suggests that elevated suPAR levels might reflect compromised cerebrovascular health in MA.

Alternatively, increased circulating suPAR levels might be related to CSD but could have remained undetected in our sample due to the long plasma half-life and the mean number of monthly aura days being 4 in our MA population. In this scenario, repeated CSD events cause increased endothelial dysfunction, supported by pre-clinical data,^25^ thereby increasing the risk of ischaemic stroke. Further studies, possibly employing a within-subjects design, are needed to ascertain the relationship of circulating suPAR levels with days until next aura attack and days since last aura attack.

### Strengths and limitations

The strengths of this study lie in the meticulous use of in-person semi-structured interviews and a large sample size of adult participants with migraine. In addition, standardized protocols were used for sampling and processing of blood. Nonetheless, several limitations warrant mention. We selected antecubital blood sampling for its minimally invasive nature and suitability for large-scale studies, though fluids like external jugular venous blood or CSF might better reflect suPAR levels in brain and meningeal tissues. The cross-sectional design precludes us from examining alterations in plasma suPAR levels within persons with migraine during ictal and inter-ictal phases. Furthermore, due to practical constraints, blood samples were not systematically collected during specific migraine phases. However, our large sample size allowed robust analyses of suPAR levels across both the ictal and inter-ictal phases, and we found no evidence to suggest an influence from migraine headache. There were also slight discrepancies in age between participants with migraine and healthy controls, as well as a skewed distribution of asthma, and intake of triptans ≤72 h prior to blood sampling between the MA and MO groups. These variations in covariates pose another limitation, particularly since suPAR levels are known to increase with older age and have been found to be elevated in individuals with asthma compared with healthy controls.^61,62^ As such, we incorporated these variables, among other relevant covariates, in our statistical analyses. Lastly, the predominance of White participants, primarily enrolled from a tertiary care unit, might introduce sampling bias. Caution is thus warranted when extrapolating our findings to other racial groups and the broader migraine population.

## Conclusion

Our findings demonstrate elevated plasma suPAR levels in participants with MA suggesting the presence of low-grade chronic inflammation. Further insights into the molecular mechanisms linking inflammation and MA could pave the way for developing targeted therapies.

## Supplementary Material

fcae475_Supplementary_Data

## Data Availability

Upon reasonable request, the corresponding author can provide data and materials that were used and analysed for the purposes of this study.
