# Supplementary material for: Association of plasma soluble urokinase plasminogen activator receptor concentrations and migraine with aura: a REFORM study
Source: Brain Commun. 2025 Feb 17;7(1):fcae475. doi: 10.1093/braincomms/fcae475 (PMC11831075; doi:10.1093/braincomms/fcae475)
Supplement: fcae475_Supplementary_Data [file fcae475_supplementary_data.pdf]

Supplementary Material

Tesfay B, et al. Association of Plasma suPAR Concentrations and Migraine with Aura: A REFORM Study

Supplementary Table 1. Overview of statistical analyses

Supplementary Table 2. Plasma suPAR levels for participants with migraine and healthy controls

Supplementary Table 3. Estimated relative differences in plasma suPAR in exploratory analyses

Supplementary Table 4. Univariate and multivariable linear regression analyses of covariates

Supplementary Figure 1. Plasma suPAR levels in exploratory outcomes

This supplementary material has been provided by the authors to give readers additional information about their work.

Supplementary Table 1. Overview of statistical analysis

Comparison	Method of analysis
Data distribution	Histogram and QQ-plots
Demographics and characteristics across groups	Unpaired t-test,<br>Wilcoxon rank-sum test or<br>Pearson's χ^2-test
Association between suPAR levels and the following variables:<br>- Age<br>- Sex<br>- BMI<br>- Smoking status<br>- Ongoing or history of autoimmune disorders<br>- Other comorbidities<br>- Current use of preventive migraine medication<br>- Use of NSAIDs, triptans or combination drug ≤ 72 hours prior to blood sampling	Generalized linear model,<br>Univariate model
Variables from Univariate model with *P* value <.10	Multivariable linear regression model
Plasma suPAR levels	Linear regression adjusted for age, sex, BMI, smoking status (smoker/non-smoker).
Correlation between plasma suPAR levels and (1) mean number of MMD, and (2) mean number MMD with both headache and aura, and (3) mean monthly days with acute headache medication use.	Spearman's rank correlation coefficient
Multiple hypothesis testing	Bonferroni method
Abbreviations: BMI, Body mass index; MMD, monthly migraine days; suPAR, soluble urokinase-type plasminogen activator receptor.	

Supplementary Table 2. Plasma suPAR levels for participants with migraine and healthy controls

	Participants (n)	Median (IQR)	Mean (± SD)
**Overall**			
All participants with migraine	634	2.50 (2.15-2.95)	2.59 (0.69)
Healthy controls	154	2.37 (2.02 - 2.78)	2.44 (0.56)
**History of aura**			
Migraine with aura	188	2.59 (2.19-3.03)	2.68 (0.52)
Migraine without aura	446	2.45 (2.13-2.90)	2.55 (0.66)
**Headache frequency**			
Chronic migraine	412	2.50 (2.14-2.95)	2.60 (0.68)
Episodic migraine	222	2.49 (2.17-2.92)	2.58 (0.71)
**Headache status**			
Migraine headache	285	2.50 (2.14-2.97)	2.62 (0.72)
Non-migraine headache	118	2.41 (2.17-2.84)	2.56 (0.73)
Headache-free	227	2.52 (2.17-2.93)	2.58 (0.63)
**Concurrent preventive medication**			
Chronic migraine	193	2.54 (2.16-3.00)	2.64 (0.75)
Episodic migraine	125	2.46 (2.21-2.90)	2.61 (0.76)
**No concurrent preventive medication**			
Chronic migraine	219	2.49 (2.13-2.93)	2.57 (0.62)
Episodic migraine	97	2.51 (2.07-2.96)	2.54 (0.63)

Supplementary Table 2. Plasma suPAR levels for participants with migraine and healthy controls (Continued)

	Participants (n)	Median (IQR)	Mean (± SD)
**Medication overuse headache**			
MOH	220	2.52 (2.21-2.97)	2.65 (0.72)
Non-MOH	414	2.48 (2.12-2.93)	2.56 (0.68)
**Abbreviations**: MOH, medication-overuse headache			

Supplementary Table 3. Estimated relative differences in plasma suPAR in exploratory analyses

	Unadjusted model		Adjusted model[a]	
	**Estimated relative difference (%; 95% CI)**	***P* value**	**Estimated relative difference (%; 95% CI)**	***P* value**
**Preventive medication use**				
**Chronic migraine**				
Preventives *vs* no preventives	1.8 (-3.0 to 6.7)	.45	1.0 (-4.6 to 6.8)	>.99
Preventives *vs* HC	6.7 (1.4 to 12.0)	**.013**	4.7 (-1.7 to 11.5)	.24
No preventives *vs* HC	4.9 (-0.3 to 10.0)	.063	3.7 (-0.2 to 10.2)	.46
**Episodic migraine**				
Preventives *vs* no preventives	2.0 (-4.6 to 8.6)	>.99	2.1 (-5.5 to 10.2)	>.99
Preventives *vs* HC	5.6 (-0.2 to 11.4)	.18	3.4 (-3.5 to 10.8)	.74
No preventives *vs* HC	3.6 (-2.7 to 9.9)	.79	1.3 (-5.9 to 9.1)	>.99
**Medication-overuse headache**				
MOH *vs* non-MOH	3.3 (-0.8 to 7.4)	.36	0.4 (-4.4 to 5.4)	>.99
MOH *vs* HC	7.5 (2.3 to 12.7)	**.014**	3.9 (-2.3 to 10.6)	.40
Non-MOH *vs* HC	4.2 (-0.4 to 8.9)	.22	3.6 (-1.9 to 9.4)	.38

Abbreviations: MOH, Medication-overuse headache; HC, Healthy controls.
[a] Adjusted for age, sex, body mass index, and smoking.
P values <0.05 before and after adjustment using the Bonferroni method are highlighted in bold.

Supplementary Table 4. Univariate and multivariable linear regression analyses of covariates

	Univariate		Multivariate	
	**Estimate (95% CI)**	***P* value**	**Estimate (95% CI)**	***P* value**
**Demographics**				
Age	1.004 (1.003 – 1.006)	**<.001**	1.004 (1.003 – 1.006)	**<.001**
Female sex	1.071 (1.003 – 1.144)	**.041**	1.101 (1.034 – 1.172)	**.003**
BMI	1.012 (1.008 – 1.016)	**<.001**	1.011 (1.007 – 1.015)	**<.001**
Active smoking	1.085 (1.015 – 1.159)	**.016**	1.095 (1.028 – 1.167)	**.005**
**Migraine characteristics**				
Migraine aura	1.054 (1.009 – 1.102)	**.019**	1.044 (1.001 – 1.088)	**.046**
MOH	1.033 (0.991 – 1.078)	.13		
Ictal status (migraine vs pain-free)	1.011 (0.967 – 1.057)	0.63		
Ictal status (non-migraine vs pain-free)	0.984 (0.930 – 1.041)	.57		
MMD	1.002 (0.999 – 1.004)	.53		
MMD with headache and aura	1.002 (0.995 – 1.010)	.52		
**Concomitant treatment**				
Migraine preventives	1.018 (0.978 – 1.060)	.38		
NSAID within 72 hours	1.023 (0.958 – 1.092)	.50		
Triptans within 72 hours	1.003 (0.960 – 1.048)	.90		
Statins	1.080 (0.977 – 1.193)	.13		

Supplementary Table 4. Univariate and multivariable linear regression analyses of covariates (Continued)

	**Univariate**		**Multivariate**	
	**Estimate (95% CI)**	***P* value**	**Estimate (95% CI)**	***P* value**
**Comorbidities**				
Daily neck pain (≥ 3 months)	1.004 (0.950 – 1.063)	**0.88**		
Daily low back pain (≥ 3 months)	1.136 (1.062 – 1.215)	**<.001**	1.077 (1.008 – 1.151)	**.028**
Cancer	1.051 (0.959 – 1.153)	.28		
Other cardiovascular conditions	1.069 (0.983 – 1.162)	.12		
Hypertension	1.103 (1.035 – 1.177)	**.003**	1.025 (0.961 – 1.092)	.46
Asthma	1.004 (0.941 – 1.072)	.90		
Autoimmune disorder	0.999 (0.937 – 1.066)	.98		
Anxiety	1.040 (0.972 – 1.113)	.25		
Depression	1.090 (1.019 – 1.166)	**.012**	1.048 (0.982 – 1.118)	.16

Abbreviations: BMI, Body mass index; CI, Confidence interval; MMD, Mean number of monthly migraine days; NSAID, Non-Steroidal Anti-Inflammatory Drugs. *P* values <.05 before and after adjustment using the Bonferroni method are highlighted in bold.

Supplementary Figure 1. Plasma suPAR levels in exploratory outcomes

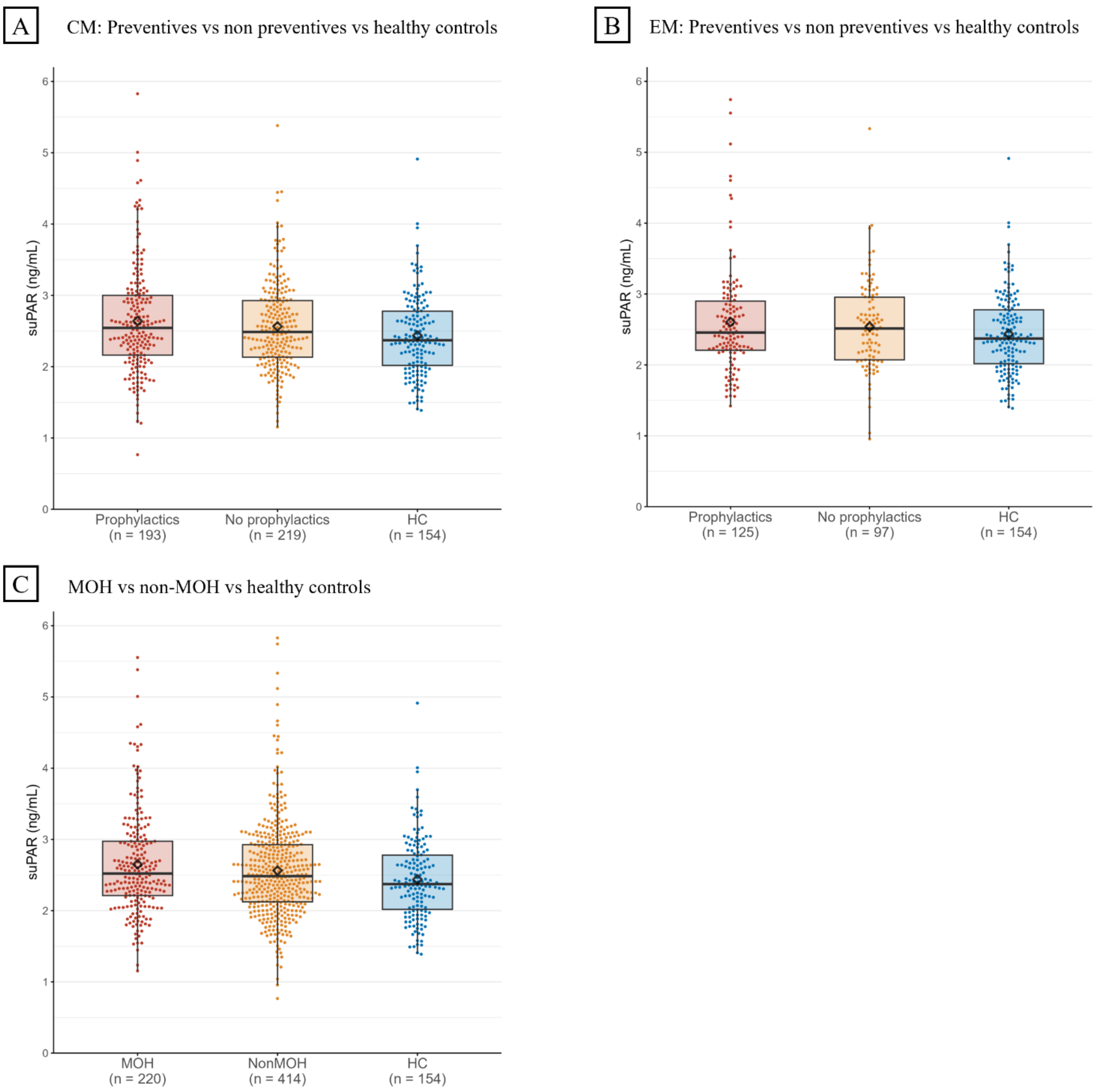

Plasma soluble urokinase-type plasminogen activator receptor (suPAR) levels in (A) participants with episodic or (B) chronic migraine on preventive migraine medication(s), compared with those without preventive migraine medication, and healthy controls (HC); and (C) participants with MOH (medication-overuse headache) compared with those without MOH, and HC. Box plot representation of median (horizontal bar), IQR (hinges), and 1.5 x IQR (whiskers). Individual measurements of suPAR are represented by dots. There were no statistically significant differences (*P* <.05) between the subgroups.
